# An Insidious Case of Severe Acquired Factor VIII Inhibitor

**DOI:** 10.7759/cureus.48467

**Published:** 2023-11-07

**Authors:** Falguni Patel, Kimberly Boldig, Jennifer Miatech, Pramod Reddy

**Affiliations:** 1 Internal Medicine, University of Florida College of Medicine, Jacksonville, USA; 2 Hematology and Oncology, University of Florida College of Medicine, Jacksonville, USA

**Keywords:** coagulation factor viii, hemorrhagic bleed, acquired coagulation disorders, acquired factor viii deficiency, acquired factor inhibitor

## Abstract

Acquired hemophilia occurs when neutralizing antibodies inhibit the activity of coagulation factors, commonly occurring with factor VIII. Most cases are idiopathic; however, autoimmune diseases, certain medications, and malignancies can predispose patients to the development of these inhibitors. Moreover, the initial presentation of the disease is more often catastrophic bleeding, with ecchymosis or mucosal bleeding being less common. This case report outlines an incidental finding of a severe factor VIII inhibitor (with 0% activity) with non-catastrophic bleeding at presentation in the setting of potential lymphoma. Subsequently, the patient was treated with recombinant factor VIIa and immunosuppression with steroids. The case sheds light on the benign presentation of a rapidly fatal disease, thus necessitating urgent and rapid identification. Given the insidious presentation, further research is required on the various factor inhibitors to reduce health costs and improve mortality.

## Introduction

Acquired inhibitors of coagulation, also known as acquired hemophilia, refer to autoimmune conditions in which various antibodies can inhibit the function of coagulation factors or increase the body’s clearance of said factors [1]. Various disease states can predispose patients to these inhibitors, including autoimmune disease and malignancy. There is also an increased risk during the postpartum period of pregnancy. Reports of drug-induced inhibitors have also been reported with clopidogrel, phenytoin, and penicillins [2]. Despite the numerous etiologies, most cases of acquired hemophilia are idiopathic, approximately 50% of cases [3]. Overall, acquired inhibitors of coagulation are rare in the general population and can occur against all coagulation factors.

The incidence of disease increases with age and more commonly occurs in patients over 65 years of age with immune-altering diseases. The initial presentation of the disease varies from non-traumatic ecchymosis to catastrophic bleeding. Approximately 87% of cases initially present with major bleeding, of which 22% succumb to bleeding [4]. The severity of the disease is often classified using factor activity level [5]. As most of these inhibitors are commonly against factor VIII, a prolonged activated partial thromboplastin (aPTT) can be found as an initial laboratory finding. In 10% of the cases, the aPTT is the only presenting symptom; therefore, it should always be investigated further [6]. Signals of disease may also include non-traumatic or spontaneous ecchymosis, mucosal bleeding, retroperitoneal bleeds, or post-surgical bleeding. After the appropriate diagnostic tests are completed, prompt treatment should include stabilization of bleeding and elimination of inhibitors through immunosuppressive therapy [6]. The following case represents an insidious presentation of this potentially life-threatening and uncommon disease.

## Case presentation

A 68-year-old male with a medical history of hypertension, stage 3 chronic kidney disease, tobacco abuse disorder, and chronic venous stasis presented inpatient for an elective angiogram for further evaluation of venous stasis ulcers. Prior to the procedure, the patient was found to have an isolated aPTT. The hematology service was consulted for surgical clearance. The patient endorsed a one-month history of diffuse bruising without significant trauma, an unintentional 15-pound weight loss over the past three months, and gum bleeding. He described dentition trauma, which resulted in mucosal bleeding for about two weeks and the formation of blood clots. The patient denied a history of excessive bleeding or bruising prior to this event. The patient did not maintain routine medical care or cancer screenings. The patient's family history was negative for hemophilia, other bleeding and coagulation disorders, and malignancies. The patient denied a history of excessive bleeding with prior surgical procedures. Social history included chronic tobacco use (40 pack-year) and denial of alcohol and illicit drug use.

On the initial exam, the patient was hemodynamically stable. The physical exam was remarkable for a large left upper extremity ecchymosis, with smaller ecchymosis present diffusely throughout the body. Left cervical painless lymphadenopathy and hepatomegaly were appreciated. The remainder of the physical exam was unremarkable.

Initial diagnostics were significant for elevated aPTT (107 seconds), confirmed with a repeat value and normal prothrombin time (PT) of 11.6 seconds and an international normalized ratio (INR) of 1. Labs were significant for stable anemia of chronic disease with hemoglobin 10.2 g/dL, within the patient's baseline. Given the pronounced lymphadenopathy, A computed tomography (CT) of the soft tissue neck was obtained, which demonstrated a 10mm left cervical 2A lymph node with scattered subcentimeter lymph nodes throughout. Given unintentional weight loss and lymphadenopathy, a CT of the chest, abdomen, and pelvis was obtained for concern for malignancy. Imaging showed no evidence of a frank intra-thoracic, intra-abdominal, or intrapelvic mass. A mixing study was performed and resulted in elevated aPTT, signifying a factor inhibitor as opposed to a factor deficiency. Pertinent labs returned, demonstrating factor VIII activity as 0% and factor IX as reduced. von Willebrand (vWF) deficiency was ruled out given elevated vWF antigen (223.8%) and activity (197.8%). Antiphospholipid (APL) syndrome was ruled out given <9 APL U/mL anticardiolipin IgG, <9 GPI IgG units for beta-2 glycoprotein IgG, and a normal dilute Russell venom viper time (DRVVT) of 35.9 seconds. Furthermore, the hexagonal phase phospholipid neutralization assay returned elevated aPTT, further disproving anti-phospholipid syndrome. Table 1 presents further lab values.

**Table 1:** Pertinent initial lab values

	Reference range	Laboratory value
White blood cells (WBC)	4.5-11 thou/mm3	5.74 thou/mm3
Hemoglobin (Hgb)	14-18 g/dL	10.2 g/dL
Hematocrit (Hct)	40-54 %	30.2 %
Mean corpuscular volume (MCV)	82-101 fL	86.3 fL
Platelets	140-440 109/L	236 109/L
Ferritin	30-400 ng/mL	2,125 ng/mL
Iron	32-159 µg/dL	21 µg/dL
Total iron binding capacity (TIBC)	261-390 mcg/dL	194 mcg/dL
Transferrin	200-360 mg/dL	153 mg/dL
Activated partial thromboplastin time (aPTT)	25-27 seconds (sec)	107 sec
Protime (PT)	9.4-12.5 sec	11.6 sec
International normalized ratio (INR)	0.8-1.1	1
Dilute viper venom time (DVVT)	0-47.0 sec	35.9 sec
Mixing study	0-40.5 sec	81.4 sec
Hexagonal phase phospholipid	0-11 sec	20 sec
Factor VIII activity	55-200%	0%
Factor IX activity	65-150%	60%
Factor XI activity	60-150%	79%
Factor XII activity	50-150%	78%
Anticardiolipin immunoglobulin A (IgA)	0-11 APL U/mL	<9 APL U/mL
Beta-2 glycoprotein immunoglobulin G (IgG)	0-20 GPI IgG	<9 GPI IgG
von Willebrand activity	40-163%	197.80%
von Willebrand antigen	42-176%	223.80%

Additionally, further infectious and autoimmune workups, including rheumatoid factor, antinuclear antibody, human immunodeficiency virus, and hepatitis panel, resulted in negative results.

Given the presence of lymphadenopathy without evidence of an alternative factor VIII inhibitor inciter, lymphoma was of concern. Plans for an excisional lymph node biopsy were made once the risk of catastrophic bleeding was minimized. The patient was started on immunosuppressive therapy with methylprednisolone 1 mg/kg and gastrointestinal prophylaxis to combat the inhibitor. During the hospital course, the patient was noted to have a 2 g/dL decrease in hemoglobin with evidence of diffuse ecchymosis, and therefore, recombinant factor VIII (rFVIII) was also given at 45 mcg/kg to aid with bleeding as the patient developed a two-point drop in hemoglobin over his admission. Despite a hemoglobin decrease, the patient remained hemodynamically stable and asymptomatic, with no sign of overt hemorrhagic diathesis. The patient received three days of treatment, with daily factor VIII activity values remaining at 0%. Treatment was ended prematurely as the patient left against medical advice. An outpatient check-in phone call was completed two weeks after admission, and the patient stated he was “feeling well”. 

## Discussion

Factor VIII is an important part of the coagulation cascade. Deficiency, or the presence of an inhibitor, most commonly results in catastrophic bleeding. Factor VIII plays an important role in increasing the efficiency of factor IXa in the activation of factor X [6,7]. Congenital hemophilia is known as a genetic deficiency of factor VIII. Acquired hemophilia A is the development of factor VIII inhibitor [7]. Congenital hemophilia may present with hemarthrosis. However, acquired hemophilia is more likely to present with purpura and soft tissue bleeding [7]. The incidence of acquired hemophilia has been reported to affect 1.2-1.48 cases per million per year [8,9]. The mortality rate has been reported to be between 3% and 9%, with improved therapeutic options [10]. Most cases are idiopathic, although it is noted to be associated with malignancy or autoimmune disorders [11]. Most patients present with bleeding (94.6%) ranging from mild ecchymosis to catastrophic hemorrhage with possible subcutaneous, gastrointestinal, and muscular tissue involvement [12,13]. Laboratory findings include prolonged aPTT, normal PT, thrombin time, and platelet count [7]. A mixing study may be used to differentiate from other disease states and confirm the presence of an inhibitor [7]. Testing to eliminate heparin contamination and lupus anticoagulant as possible causes of acquired hemophilia allows confirmation of a factor VIII inhibitor [7].

Treatment decisions are difficult with acquired hemophilia because the factor inhibitor levels and degree of bleeding at presentation are incredibly variable and not correlated. Neither factor VIII levels nor inhibitor levels are effective measures to predict bleeding intensity [13]. Additionally, because of these factors, no evidence-based treatment recommendation exists. Treatment options for acquired hemophilia consist of activated prothrombin complex concentrate (aPTT) or recombinant activated factor VII (rVIIa) [10, 13, 14]. Both treatments allow the bypass of factor VIII to augment the subsequent coagulation cascade [13, 14]. Treatment of the inhibitor itself occurs with steroids, steroids and cyclophosphamide, or rituximab as second-line therapy [9]. Observation may be appropriate with a presentation of subcutaneous bleeding [10]. However, conflicting research states that factor VIII concentrates, desmopressin, or plasmapheresis may be considered for minor bleeding [15]. About 10% to 20% of patients will relapse [10, 11]. The European Acquired Hemophilia (EACH2) trial reports an 18% relapse rate in patients treated with corticosteroids [3]. Therefore, it is recommended to obtain aPTT and factor VIII levels monthly for the first six months [10]. Importantly, patients remain at risk for severe bleeding until the inhibitor is eradicated, despite the initial factor VIII level, inhibitor level, and degree of bleeding [3].

This case describes an atypical presentation of a rare coagulation disorder that may lead to catastrophic bleeding and death. Our patient's presentation of an abnormal aPTT lab allowed prompt assessment and diagnosis. This diagnosis was seemingly idiopathic. However, further evaluation of lymphadenopathy was postponed due to the high bleeding risk, which limited the complete evaluation of the etiology. Our patient presented with ecchymosis and a factor VIII level of 0%, classified as a severe disease [5]. This displays how patient presentation and inhibitor levels are not congruent. Although our patient presented with subcutaneous bleeding, he described recent mucosal bleeding prior to presentation. Although no treatment guidelines exist for acquired hemophilia, research may indicate conservative treatment with monitoring for minor bleeds, such as subcutaneous bleeding [16]. However, the patient subsequently developed a hemoglobin drop during hospitalization without signs of active bleeding, creating a more ominous concern of catastrophic bleeding. This brings to light the difficulty in establishing which patients are at high risk for catastrophic bleeding. Unfortunately, patient factors limit completing treatment.

While the patient's diagnosis was incomplete, the evaluation of our patient for possible hematologic malignancy was important. Acquired hemophilia is commonly associated with hematologic malignancies [15]. This stresses the importance of finding the etiology of the factor inhibitors, as they can be the presenting symptom of a more deadly disease. While acquired factor inhibitors are an overall rare occurrence, the common etiology of hematological malignancies should be ruled out [17].

## Conclusions

Acquired inhibitors of coagulation are often associated with catastrophic bleeding and insidious onset, with common presentations of prolonged aPTT and subtle ecchymosis. In the case presented, the patient presented with minor symptoms in the presence of severe disease (factor VIII activity: 0%). Given the decrease in hemoglobin levels as well as the potential for catastrophic bleeding, the decision to treat with recombinant factor VIIa and immunosuppression was made. The case was limited by patient cooperation as well as the inability to completely work up the etiology with a lymph node biopsy. The case identifies the broad presentation and laboratory findings that may exist with the limited treatment guidelines that are currently present. A thorough evaluation of the underlying etiology of acquired hemophilia is important; however, certain procedures are often limited in the setting of increased bleeding risk. Further research is warranted to continue to follow these patients to describe subsequent bleeding events, response to treatment, outcome, remission time, recurrence, and identification of the etiology of the disease. Further research is needed to fully assess this life-threatening disease.
